# miRNA Reference Genes in Extracellular Vesicles Released from Amniotic Membrane-Derived Mesenchymal Stromal Cells

**DOI:** 10.3390/pharmaceutics12040347

**Published:** 2020-04-11

**Authors:** Enrico Ragni, Carlotta Perucca Orfei, Antonietta Rosa Silini, Alessandra Colombini, Marco Viganò, Ornella Parolini, Laura de Girolamo

**Affiliations:** 1 IRCCS Istituto Ortopedico Galeazzi, Laboratorio di Biotecnologie Applicate all’Ortopedia, Via R. Galeazzi 4, I-20161 Milano, Italy; carlotta.perucca@grupposandonato.it (C.P.O.); alessandra.colombini@grupposandonato.it (A.C.); marco.vigano@grupposandonato.it (M.V.); laura.degirolamo@grupposandonato.it (L.d.G.); 2 Centro di Ricerca E. Menni, Fondazione Poliambulanza Istituto Ospedaliero, Via Bissolati 57, I-25124 Brescia, Italy; antonietta.silini@poliambulanza.it (A.R.S.); ornella.parolini@poliambulanza.it (O.P.); 3 Department of Life Science and Public Health, Università Cattolica del Sacro Cuore, Largo F. Vito 1, I-00168 Rome, Italy

**Keywords:** mesenchymal stromal cells, placenta, amniotic membrane, extracellular vesicles, miRNAs, reference genes, tendinopathy, regenerative medicine, nanocarriers, delivery platforms

## Abstract

Human amniotic membrane and amniotic membrane-derived mesenchymal stromal cells (hAMSCs) have produced promising results in regenerative medicine, especially for the treatment of inflammatory-based diseases and for different injuries including those in the orthopedic field such as tendon disorders. hAMSCs have been proposed to exert their anti-inflammatory and healing potential via secreted factors, both free and conveyed within extracellular vesicles (EVs). In particular, EV miRNAs are considered privileged players due to their impact on target cells and tissues, and their future use as therapeutic molecules is being intensely investigated. In this view, EV-miRNA quantification in either research or future clinical products has emerged as a crucial paradigm, although, to date, largely unsolved due to lack of reliable reference genes (RGs). In this study, a panel of thirteen putative miRNA RGs (let-7a-5p, miR-16-5p, miR-22-5p, miR-23a-3p, miR-26a-5p, miR-29a-5p, miR-101-3p, miR-103a-3p, miR-221-3p, miR-423-5p, miR-425-5p, miR-660-5p and U6 snRNA) that were identified in different EV types was assessed in hAMSC-EVs. A validated experimental pipeline was followed, sifting the output of four largely accepted algorithms for RG prediction (geNorm, NormFinder, BestKeeper and ΔCt method). Out of nine RGs constitutively expressed across all EV isolates, miR-101-3p and miR-22-5p resulted in the most stable RGs, whereas miR-423-5p and U6 snRNA performed poorly. miR-22-5p was also previously reported to be a reliable RG in adipose-derived MSC-EVs, suggesting its suitability across samples isolated from different MSC types. Further, to shed light on the impact of incorrect RG choice, the level of five tendon-related miRNAs (miR-29a-3p, miR-135a-5p, miR-146a-5p, miR-337-3p, let-7d-5p) was compared among hAMSC-EVs isolates. The use of miR-423-5p and U6 snRNA did not allow a correct quantification of miRNA incorporation in EVs, leading to less accurate fingerprinting and, if used for potency prediction, misleading indication of the most appropriate clinical batch. These results emphasize the crucial importance of RG choice for EV-miRNAs in hAMSCs studies and contribute to the identification of reliable RGs such as miR-101-3p and miR-22-5p to be validated in other MSC-EVs related fields.

## 1. Introduction

Current knowledge on mesenchymal stromal cells (MSCs) as future medicinal products in the field of regenerative medicine sheds light on the crucial role of their secreted factors and extracellular vesicles (EVs) to stimulate tissues and cells. In particular, by avoiding the concerns of using live and replicating cells that may undergo mal-differentiation or mutation, the idea of EVs as therapeutics is being explored, since they can mimic several biological actions of MSCs [1] through embedded lipids, proteins, mRNAs and miRNAs [2,3], therefore acting as biological nanocarriers. Even more intriguing, MSC-EVs may be engineered by either exogenous load [4] or reprogramming of the secreting cells [5,6] to shuttle and deliver specific and therapeutic molecules, such as miRNAs or mRNAs, eventually driving the focused influence on target cells [7]. As an example, in the orthopedic field, which is one of the most actively studied areas in regenerative medicine, miRNA-enriched MSC-EVs were shown to promote cartilage regeneration [5,6] by modulating ECM production. Similarly, in adipose-derived MSCs (ASCs), inflammatory priming of cells was able to modulate the EVs incorporation of miRNAs involved in both cartilage homeostasis and macrophage M2-polarization [8,9]. Thus, there is a growing interest in both the discovery and characterization of new MSC-EVs for their future use as natural or engineered delivery platforms in regenerative approaches.

In this frame of active investigation of new players, among the different tissues that have been studied for MSC and MSC-EV isolation, basic research fingerprinting and eventual clinical use, the amniotic membrane from human term placenta has gained interest as privileged source for several reasons: it is discarded after birth thus is considered biological waste, and its procurement does not require an invasive procedure, nor does it pose any ethical issues. Moreover, and most importantly, it is rich in stromal cells (hAMSCs) with immunomodulatory and regenerative properties [10]. Due to these features, in different preclinical inflammatory disease models, hAMSCs and their conditioned medium (CM-hAMSC) have been successfully exploited, including for lung [11,12,13,14] and liver fibrosis [15], wound healing [16,17,18], collagen-induced arthritis [19,20], multiple sclerosis [19], inflammatory bowel disease, colitis [21], sepsis [19], traumatic brain injury [22], and Huntington’s disease [23]. Accordingly, in vitro, we and others have demonstrated that hAMSCs and CM-hAMSC suppress the proliferation, inflammatory cytokine production, and functions of T lymphocytes [24,25], monocytes [18], dendritic cells [26], macrophages [18], and natural killer cells [27], while inducing a phenotype and functional switch of monocytes toward macrophages with anti-inflammatory pro-regenerative M2-like features [18,25], supporting the expansions of regulatory T cells [24,25].

Moreover, in the field of regenerative medicine, both hAMSCs and CM-hASMC have demonstrated promising results by restoring tissue damage or promoting healing, as has emerged in orthopedic applications [28,29,30]. The whole CM was able to enhance collagen production and biomechanical properties at the site of Achilles tendon transection in rats [31,32]. Moreover, a pioneer report in the equine model showed in vitro that purified AMSC-derived EVs are key players in healing of tendon lesions, exerting anti-inflammatory effects on tenocytes [33]. These effects were postulated to be at least partially dependent on the profiles of embedded miRNAs, as subsequently described [34], shining a light on AMSC-EV characterization and conveyed miRNA fingerprints.

Therefore, purified and clinical-grade hAMSC-EVs may be an innovative cell-based delivery platform to shuttle therapeutic miRNAs, both naturally or exogenously loaded, to facilitate tissue repair and inflammation control during pathological processes. In this perspective, accurate miRNA quantification is a major challenge, largely depending on the choice of a proper normalization strategy [35]. While the global mean normalization has proven to be highly sensitive and accurate for whole-genome RT-qPCR-based miRNA profiling [36], the selection of miRNA reference genes (RGs) for single or few assays, as needed for both basic research and release tests for clinical-grade products, remains a challenge. To date, no universal miRNA normalizers have been defined in cell extracts, since those identified are very often tissue or cell-type exclusive. Regarding EVs, the situation is even more difficult, and the establishment of reliable RGs is mandatory. Recently, our group proposed an in-silico pipeline to identify new miRNA RGs in EVs isolated from ASCs [37,38]. Several applets (geNorm, NormFinder, BestKeeper and ΔCt method) [39,40,41,42] were used to score the stability of a selected panel of miRNAs to identify those resulting as more stable across either donors or divergent culturing conditions. This process resulted in a mandatory step both for reliable basic research studies and to efficiently characterize the potency of a future therapeutic product, by assessing and comparing the precise amount of MSC-EVs active factors, such as naturally occurring or artificially modulated miRNAs.

To increase the knowledge in the MSC field, in the present study we characterized the stability of a wide panel of putative miRNA RGs in hAMSC-EVs to provide a valuable tool in order to fingerprint these microparticles. In particular, the crucial impact of RG choice has been emphasized by highlighting the dramatic differences in the amount of evaluation of few and unrelated tendon-associated miRNAs, chosen as examples due to the emerging role of small RNA gain or loss in tendon homeostasis and tendinopathy development [43,44,45]. In general, the herein-proposed results will be helpful to envision hAMSC-EVs as delivery platforms in the broader context of regenerative-medicine-based approaches, where immunomodulation and regeneration are needed.

## 2. Materials and Methods

### 2.1. Ethics Statement

Human term placentas (N = 3) were recovered from healthy women after vaginal delivery or caesarean section at term. The study was conducted in accordance with the Declaration of Helsinki, and samples were collected after obtaining informed written consent according to the guidelines set by the Comitato Etico Provinciale of Brescia, Italy number NP 2243 (19/01/2016).

### 2.2. hAMSC Isolation and Expansion

Placentas were processed immediately after collection and cells were isolated and directly used. Specifically, human amniotic mesenchymal stromal cells (hAMSCs) were obtained from the mesenchymal region of the amniotic membrane as previously described [46]. hAMSCs were maintained in CHANG C Medium (Irvine Scientific, Irvine, CA, USA) at 37 °C, 5% CO_2_, and 95% humidity. Experiments were performed with cells at passages 2 for EV collection and passage 3 for flow cytometry.

### 2.3. hAMSC Characterization

Flow cytometry analysis was performed with following antibodies: CD90-FITC (REA897), CD73-PE (REA804), CD44-PEVIO770 (REA690), CD34-PE (AC136), CD45-PEVIO770 (REA747) and CD31-PERCPVIO700 (REA730) (Miltenyi, Bergisch Gladbach, Germany). Staining was performed at 4 °C for 30 min in the dark following dilution suggested by manufacturer. A CytoFlex (Beckman Coulter, Fullerton, CA, USA) flow cytometer was used collecting a minimum of 30,000 events.

### 2.4. hAMSC-EV Isolation and Characterization

To obtain the cell culture medium, hAMSCs at 90% confluency were washed three times with PBS and CHANG B medium without supplement added (12 mL per T175 cell culture flask). After 48 h, culture supernatant was collected and serially centrifuged at 4 °C to eliminate debris and floating cells at 376× *g* for 15 min, 1000× *g* for 15 min, 2000× *g* for 15 min and twice at 4000× *g* for 15 min each to remove apoptotic bodies. Eventually, supernatant was centrifuged at 100,000× *g* for 3 h at 4 °C in a 70Ti rotor (Beckman Coulter), and pellets were processed as following depending on the downstream application:

(a) Flow cytometry. Pellets were suspended in 100 μL PBS per initial 10 mL culture supernatant. EVs were further diluted 1:500 in PBS and 100 nM CFSE added for 30 min at 37 °C in the dark. Then, antibodies (Biolegend, San Diego, CA, USA) CD9-APC (HI9A), CD63-APC (H5C6) and CD81-APC (5A6) or Miltenyi CD73-PE (REA804) and CD44-PE (130-110-293) were added to 100 μL of labelled EVs following manufacturers’ instructions and incubation performed for 30 min at 4 °C in the dark. After a further 1:1 dilution with PBS, samples were analyzed with a CytoFlex flow cytometer comparing outcomes with those obtained running FITC-fluorescent beads of 160, 200, 240, and 500 nm (Biocytex, Marseille, France). Unstained EVs and clean PBS supplemented with CFSE were used as negative controls.

(b) Nanoparticle tracking analysis (NTA). hAMSCs-EVs in suspension after ultracentrifugation were 1:250 diluted in PBS and visualized by Nanosight LM10-HS system (NanoSight Ltd., Amesbury, UK). Three recordings of 60 s were performed for each EV sample. NTA software was used to analyze collected data, providing both concentration measurements and high-resolution particle size distribution profiles. To confirm lack of major protein contamination, number of particles was related to total protein amount and EV batches considered of good purity when falling in the 10^8^ to 10^10^ particle/μg protein range, as described in [47].

### 2.5. Candidate RG Selection

According to the literature, 12 miRNAs and one small RNA (U6 snRNA) were selected for stability analysis (Table 1) [8,48,49,50,51,52,53,54,55].

### 2.6. Total RNA Isolation and miRNA Profiling

After 48 hours, culture in serum-free medium, supernatants with similar hAMSC numbers (2.3 ± 0.1 × 10^6^, mean ± SEM) were 1:1 diluted in PBS and ultracentrifuged at 100,000× *g* at 9 h for 4 °C, and pellet suspended in Trizol before processing with miRNeasy and RNeasy Cleanup Kits (Qiagen, Hilden, Germany). Before RNA extraction, to evaluate and control the efficiency of RNA recovery and cDNA synthesis, samples were spiked in with exogenous *Arabidopsis thaliana* ath-miR-159a (30 pg) synthetic miRNA. ath-miR-159 specific primers are provided in the RT and PreAmp primer pools (Life Technologies, Foster City, CA, USA). cDNA for each sample was prepared by standard reverse transcription and pre-amplification steps, as previously reported [61], and stored at −20 °C. According to the manufacturer’s instructions, expression analysis with the OpenArray system (Life Technologies) was performed into 384-well OpenArray plates. Missing values and values with C_RT_ > 27 or Amp score > 1.24 were set equal to the detection limit of C_RT_ = 28 as per manufacturer’s specification, in order to avoid results that may be stochastic. The choice of C_RT_ = 28, roughly corresponding to 1 target copy with OpenArray technology and protocol and lower than usual detection limit in qRT-PCR studies, was due to the need for preamplification reaction and the low volume of the real time reaction. When in two out of three samples values were C_RT_ > 27 or Amp score > 1.24, these samples were considered as not present. Then, the mean of positive samples was used to equalize subtle differences in the amount of starting RNA. The following assays (Life Technologies) were extrapolated for stability analysis: has-miR-22-5p 002301; hsa-miR-23a-3p 000399; has-miR-29a-5p 002447; hsa-miR-221-3p 000524; hsa-miR-423-5p 002340; hsa-miR-16-5p 000391; hsa-miR-26a-5p 000405; hsa-miR-103a-3p 000439; hsa-miR-101-3p 002253; has-let-7d-5p 002283; hsa-miR-425-5p 001516; has-miR-660-5p 001515; U6 snRNA 001973. The following assays for tendon-related miRNA analysis (Table 1): has-miR-29a-3p 002112; has-miR-135a-5p 000460; hsa-miR-146a-5p 000468; has-miR-337-3p 002157; hsa-let-7a-5p 000377 [56,57,58,59,60].

### 2.7. Data Analysis

ath-miR-159 spike-in C_RT_ values resulted to be 21.87 ± 0.11 (mean ± SEM). Due to its stability, ath-miR-159 C_RT_ was used for the equalization of technical differences during the whole process. Four applets (geNorm, NormFinder, BestKeeper and ΔC_t_ method) [39,40,41,42] were used to calculate putative RG stability. Briefly, using geNorm, the C_RT_ values were transformed to quantities in a linear value using the formula 2*^Δ^*^Ct^, where *ΔC*_t_ = the minimum Ct value − Ct value of samples in the experiment. geNorm computes all possible average pairwise variations between the transformed C_RT_ values and gives a measure of the expression stability (M) of each RG. An M-value below 1.5 identifies stability. geNorm then performs stepwise exclusion of the RG with the highest M-value (least stably expressed gene) and recalculates M-values for the remaining RGs. For NormFinder, the C_RT_ value of each RG was converted to relative quantity data as described for geNorm. The algorithm establishes the ranking order of RGs based on the stability values (SV) from the combination of intragroup variation (within each sample) and intergroup variation (within each reference gene) based on C_RT_ values. It indicates that the SV ranking order corresponds to the RG stability order. The lowest stability value corresponds to the most reliable RG. The BestKeeper algorithm determines the RG ranking from the standard deviation (SD) of the descriptive statistics of C_RT_ values that are used for the pairwise correlation analyses. The ranking order of SD values corresponds to the stability order of RGs. Similarly, the lowest SD value corresponds to the most reliable RG. Eventually, the ΔC_t_ method determines the ranking order from the mean SD calculated from the intragroup ΔC_RT_ values of “pairs of RGs”. The ranking order of mean SD values corresponds to the stability order, with the lowest mean SD value corresponding to the most reliable RG. The geometric mean (geomean) of each RG ranking across the four programs was eventually determined, leading to a consensus stability score.

Principal components analysis (PCA) plots and heatmaps were generated with ClustVis package (https://biit.cs.ut.ee/clustvis/) [62]. C_RT_ values normalized both with stable miR-101-3p/miR-22-5p and unstable miR-423-5p/U6 snRNA were analyzed. Dendrograms were generated using the following settings for both row and column clustering distance and method: correlation and average, respectively.

### 2.8. Statistical Analysis

GraphPad Prism Software version 5 (GraphPad, San Diego, CA, USA) was used to perform statistical analyses. Grubb’s test was used to identify and exclude possible outliers. The comparison between the groups was performed by an using unpaired Student’s *t*-test with significance level set at *p*-value < 0.05.

## 3. Results

### 3.1. hAMSCs and EVs Characterization

**Figure 1:**
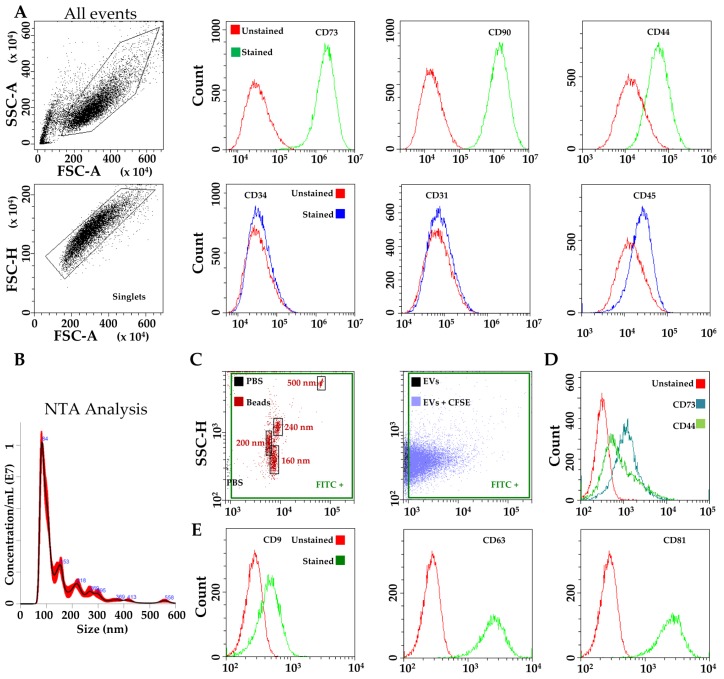
Characterization of hAMSCs and hAMSC-EVs. (**A**) Flow cytometry analysis of MSC (CD73, CD90 and CD44) and hemato-endothelial (CD31, CD34 and CD 45) markers, absence and presence respectively, confirming hAMSCs identity. Representative plots are shown: (**B**) Representative nanotracking analysis of hAMSC-EVs; (**C**) Flow cytometry of FITC-labeled nanoparticles assuring calibration of flow cytometer and comparison with CFSE-labeled hAMSC-EVs; (**D**) Presence of MSC-markers CD73 and CD44 on CFSE-labeled hAMSC-EVs. Representative plot is shown under the FITC+ gate of EVs+CFSE; (**E**) Presence of EV-markers CD9, CD63 and CD81 on CFSE-labeled hAMSC-EVs. Representative plots are shown under the FITC+ gate of EVs+CFSE.

Flow cytometry analysis was used to confirm the phenotype of hAMSCs [10]. Typical MSC cell-surface antigens, including CD44, CD73 and CD90, were highly expressed, whereas hemato-endothelial markers, such as CD31, CD34 and CD45, were not present, as previously reported [18,26] (Figure 1A).

EVs isolated from hAMSCs were first analyzed by nanoparticle tracking analysis (NTA). hAMSC-EVs were within the expected extracellular vesicle size range (mode of 83 ± 2 nm) (Figure 1B), with 80% below 200 nm, indicating enrichment in small vesicles and loss of apoptotic bodies. Flow cytometry comparing hAMSC-EVs with FITC-beads of defined size (160-200-240-500 nm) confirmed their dimensional range (Figure 1C) and the validity of the NTA measurements, which can be influenced by the residual presence of protein aggregates or lipoproteins, although analyzed batches were found to have 0.395 × 10^9^ ± 0.015 particles/μg protein (N = 3, mean ± SD), and therefore considered as depleted of major contaminants [47]. Again, EVs smaller than 200 nm resulted in the vast majority (>80% of total events). Further, CD44 and CD73, both MSC-EV-defining markers [63] and strongly positive on parental cells, were detected (Figure 1D), with CD34 again absent (data not shown). Additionally, hAMSC-EVs strongly expressed both CD63 and CD81, consistent with previously reported characteristics of EVs (Figure 1E). Eventually, CD9, another EV marker, staining gave a weak signal of the entire population (Figure 1E), similarly to vesicles derived from other MSC types [64].

### 3.2. Expression of Candidate Reference Genes

**Figure 2:**
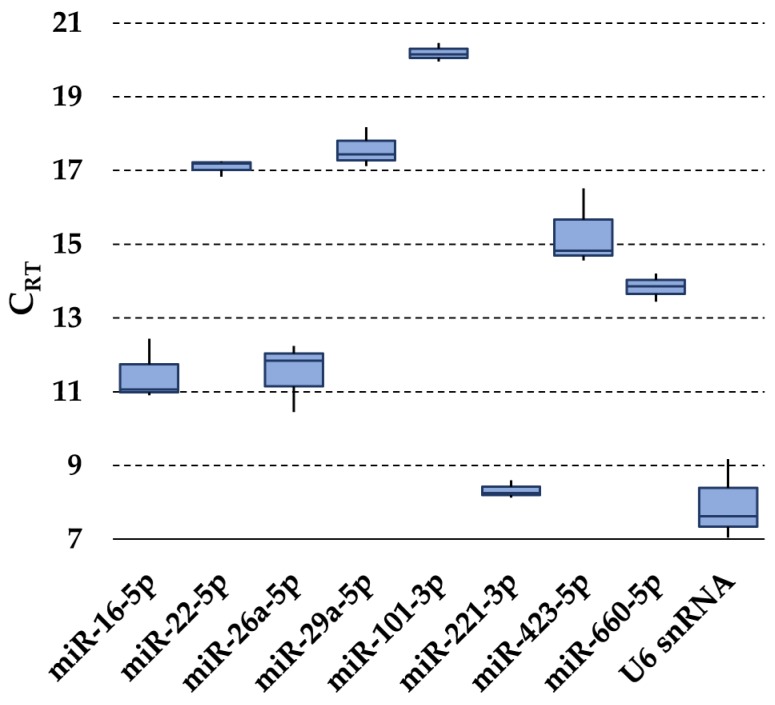
Expression of candidate RG miRNAs in hAMSC-EVs. The box plot graphs of the C_RT_ values for each RG illustrate the interquartile range (box) and median. The whisker plot depicts the range of the values.

The presence of the 13 selected RGs (Table 1) was first assessed in purified hAMSC-EVs. In all samples, let-7a-5p, miR-23a-3p, miR-103a-3p and miR-425-5p were not detected. Notably, miR-425-5p’s lack of amplification confirmed the absence of cellular contamination, since it was recently reported to be expressed in hAMSCs [65]. U6 snRNA had the highest expression, whereas miR-101-3p had the lowest (Figure 2). None of the tested candidates resided within the same gene cluster, therefore reducing the likelihood of including co-regulated miRNAs in the stability analysis [66].

### 3.3. RGs Stability Analysis

Four algorithms were run (geNorm, NormFinder, BestKeeper, and the comparative Delta Ct method) to rank the stability of the nine RGs (Table 2). Genorm identified the couple miR-22-5p/miR-101-3p (M value of 0.19) and miR-221-3p (0.28) as the most stable candidates. U6 snRNA ranked in the last position (0.95). NormFinder analysis identified miR-101-3p (SV of 0.09) as the most stably expressed RG, followed by miR-22-5p (0.10) and miR-660-5p (0.16). U6 snRNA was confirmed to be the worst performer (1.18). Using BestKeeper, miR-22-5p (SD of 0.17), miR-101-3p (0.18) and miR-221-3p (0.18) showed the lowest SD variation, while U6 snRNA the highest (0.82). Eventually, the Delta Ct method gave outcomes similar to those obtained through geNorm analysis, with the three most stable RGs being miR-101-3p (SD of 0.68), miR-22-5p (0.69) and miR-221-3p (0.74). In agreement with previous analyses, U6 snRNA resulted as the poorest candidate (1.29).

Although the four applets gave similar stability rankings, to identify a definitive and reliable hierarchy an integration and normalization of the data was mandatory. We calculated the geometric mean (geomean) of each RG weight across the four algorithms, considering the RG with the final lowest value as the most stable. Under this final analysis, miR-101-3p emerged as the best candidate (geomean of 1.32), tightly followed by miR-22-5p (1.41). Conversely, miR-423-5p (8) and U6 snRNA (9) clearly ranked last, making their choice unfavorable.

### 3.4. Impact of RGs Choice on the Quantification of Target Genes

**Figure 3:**
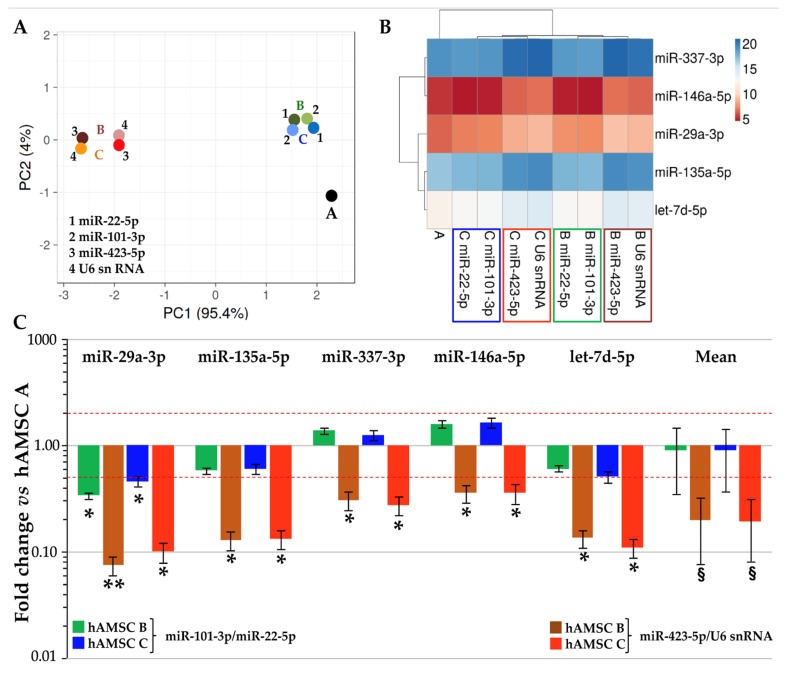
Influence of RG selection on tendon-related hAMSC-EVs miRNA profile. (**A**) through (**B**) Principal components analysis and dendrogram of the C_RT_ values of tendon-related miRNAs after stable miR-101-3p/22-5p or unreliable miR-423-5p/U6 snRNA RG normalization. (**A**)–(**C**) indicate the three donors. For the dendrogram, each row represents a miRNA and each column represents a sample. The sample clustering tree is shown at the top. The color scale shown in the map illustrates the relative expression levels of miRNAs across all samples: red shades represent high expression levels (low C_RT_) and blue shades represent lower expression levels (high C_RT_). (**C**) Effects of RG normalization on the abundance of tendon-related miRNAs differentially expressed between hAMSC-EVs samples. hAMSC A-EVs set as 1; § *p*-value < 0.1, * *p*-value < 0.05 and ** *p*-value < 0.01.

To evaluate the impact of the RG choice on miRNA expression evaluation, five candidates (miR-29a-3p, miR-135a-5p, miR-337-3p, miR-146a-5p and let-7d-5p) involved in tendon homeostasis and healing were studied in hAMSC-EVs. Amplification values (C_RT_) were compared using both stable (miR-101-3p and miR-22-5p) and unreliable (miR-423-5p and U6 snRNA) RGs, with hAMSC-EVs donor A as a touchstone. Unsupervised clustering analysis was performed to assess the effect of RG choice (Figure 3A). PCA clearly showed that the less stable RGs led to a sharper separation of hAMSC-EVs B and C samples with respect to hAMSC-EVs A. This result was further confirmed in the heat map, where both miR-423-5p and U6 snRNA were able to generate new nodes and separate clades with respect to miR-101-3p and miR-22-5p (Figure 3B). Eventually, the differential expression levels of candidate miRNAs were assessed and compared to hAMSC-EVs A, which resulted in a less clustered sample in both PCA and dendrogram. Notably, the RG choice strongly altered the correct ratios between samples leading to misleading conclusions (Figure 3C). With reliable RGs, only miR-29a-3p resulted as significantly (*p*-value < 0.05, ratio < 0.5 or > 2) reduced in both hAMSC-EVs B and hAMSC-EVs C, with no differences between them. Meanwhile, when unreliable RGs were used for comparison, all tested miRNAs resulted as significantly downregulated and biased toward a negative fold-change with respect to hAMSC-EVs A. In fact, the mean modulations of the five miRNAs were found to be 0.89 ± 0.55 and 0.89 ± 0.53 for hAMSC-EVs B and C vs. A using miR-101-3p and miR-22-5p, vs. 0.20 ± 0.12 and 0.19 ± 0.11 with miR-423-5p and U6 snRNA. Therefore, only the best RGs allowed a correct evaluation of subtle differences between donors.

## 4. Discussion

In this manuscript, the stability of putative RGs for miRNA expression analysis in EVs released from amniotic-membrane-derived MSCs was analyzed. miR-101-3p and miR-22-5p resulted as the most stable candidates, allowing for a reliable quantification of EV-embedded miRNAs, as highlighted for molecules involved and suggested as therapeutics in tendon-related pathologies.

MSC-EVs may act as biological delivery platforms able to shuttle therapeutic molecules, such as miRNAs. Further, MSC-EV cargo may be engineered by either exogenous load [4] or reprogramming of the secreting cells [5,6], making direct load comparison an imperative challenge. From this perspective, a reliable normalization approach to select stable EVs-related miRNA RGs is mandatory and, to date, only few methods have been validated. The strategy resulting in the most sensitive quantification between different samples is the normalization by global mean miRNA expression [36]. The major pitfall is the amount of required RNA, which must be high enough to score the entire or at least a large share of miRNome. Due to the reduced RNA content per EV, this would imply that a considerable portion of isolated EVs would be needed for RG identification, making the process economically unaffordable when used for clinical purposes. Thus, in view of translating basic research into potency assays to release bioactive products, it is convenient to score few miRNAs for disease-focused single assays. This implies a need for readily available and endogenous RGs [67,68,69]. In this context, U6 snRNA was recommended for miRNA quantification in pure EVs or vesicle-enriched body fluids [70,71]. Nevertheless, its suitability as EV-RG was questioned in a recent publication that analyzed cardiosphere-derived cell EVs [53]. This may be due to the mechanistically separated biogenesis of U6 snRNA, which is not processed by the spliceosome but by the Drosha complex [72]. Supporting these concerns, in hAMSC-EVs, U6 snRNA always ranked in the last position. Similarly, in ASC-EVs, U6 snRNA also scored poorly [8], suggesting its use should be avoided when EVs from mesenchymal stem cells are studied.

Under the assumption that RG miRNAs would be more indicated for miRNAs and other small RNAs, pioneering reports allowed the identification of putative miRNA RGs in EVs (Table 1). In addition, we recently identified miR-22-5p, miR-29a-5p and miR-660 as reliable RGs for EV-miRNA studies in ASCs [8]. In hAMSC-EVs, miR-101-3p and miR-22-5p resulted the best performers. Although miR-101-3p was not present in the top 20 ranking in ASC-EVs, the herein-reported results are of particular importance for much-coveted research in terms of redundancy for EV-miRNA RGs, at least in MSCs. In fact, miR-22-5p might be considered a bona fide reliable RG in studies on EVs released not only from hAMSCs, but possibly also from MSCs isolated from other sources, such as adipose tissue. Moreover, as already reported [8], miR-22-5p also resulted to be highly stable under inflammatory conditions mimicking osteoarthritis, expanding the concept of its suitability as putative EV-miRNA RG in MSCs dealing with different stimuli or pathological states. To confirm this hypothesis, future studies, focused on miR-22-5p in EVs from other MSC types and under several conditions mimicking the various physiological states of healthy/diseased individuals, will be needed to confirm at least one shared RG between MSC-EVs. In fact, universal miRNA RGs are probably very rare not only for EVs from all cell types but even for similar cells, as shown by miR-29a-5p, which was not a good performer, and miR-660-5p, which was not detected at all, or miR-101-3p, which did not reliably perform in ASC-EVs. Therefore, our results reinforce the notion that EVs have to be deeply characterized before being proposed as delivery platforms for either natural or exogenous miRNAs, and knowledge cannot be easily translated from literature, even in the same research field.

Further, in the frame of EV translation to clinical practice, another important issue is the possibility to use only a specific subset of EVs, collected by the mean of size separation or surface antigen expression. This could imply that studied miRNAs, and, in particular, miRNA RGs, have a specificity depending on EV types (small and large vesicles or apoptotic bodies). At present, the technical ability to unequivocally sort EVs and eventually fingerprint the molecular cargo is still to be developed and finely tuned for everyday use, in both research and clinics. Therefore, we propose the studied and identified miRNA RGs for the whole EV population, regardless of possible subtypes. To shed some light on association of the 12 analyzed miRNAs with small (“exosomes”) or large (“microvesicles”) vesicles, they were searched in two databases: EVmiRNA (http://bioinfo.life.hust.edu.cn/EVmiRNA/#!/) [73] and Vesiclepedia (http://microvesicles.org/index.html) [74], reporting the presence of specific miRNAs in datasets obtained from PCR or sequencing profiles of exosomes or microvesicles from different cell or tissue types. None of the miRNA RGs was found exclusively in one EV type, although the term microvesicle might indicate a large population including both small and large EVs. Therefore, the RGs proposed in this work have to be considered for the whole EV population, with the awareness that further studies will be crucial to sharply dissect miRNA and EV type association in hAMSCs and other cells or tissues.

Eventually, the crucial impact of the most reliable RG choice on miRNA abundance was studied on five tendon-related miRNAs, whose downregulation was reported to be related with tendinopathy insurgence and pathology and their supplementation envisioned as an innovative treatment. miR-29a-3p reduction leads to development of tendinopathy in both horses and humans and its reintroduction may reverse the key collagen switch that remains a tendinopathy core pathological feature [56,57]. miR-135a-5p overexpression in tendon progenitors suppresses senescence, promotes proliferation and induces migration and tenogenic differentiation [58]. In rats, the tendinopathy-associated proinflammatory cytokine IL-1β downregulates the expression of miR-337-3p, and miR-337-3p administration may accelerate tendon healing via the modulation of chondro-osteogenic and tenogenic differentiation balance of tendon progenitors [60]. In humans affected by glenohumeral arthritis or rotator cuff tears, miR-146a-5p was found to be downregulated where the severity of inflammation was greater, suggesting this molecule as a regulator of tendon inflammation [59]. In the same patients, let-7d-5p downregulation was also reported, suggesting its role in inflammatory pathways [59]. Thus, the correct evaluation of the abundance of these and other tendinopathy-related miRNAs in EVs acquires a crucial importance to compare their levels either in the same donor or between different clinical products, to choose the most appropriate EV batch as a therapeutic miRNA delivery vehicle. Of note, the use of less stable RGs led to apparent reductions for all analyzed miRNAs in hAMSC-EVs B and C vs. A, although only miR-29a-3p resulted as significantly less embedded when the correct RGs were used. Future studies on other tendon-related miRNAs, and more generally on those miRNAs involved in pathologies that are targets of regenerative medicine approaches, will be needed for a more complete picture of potentially therapeutic molecules in hAMSC-derived EVs and to develop release assays for off-the shelf clinical products. In this frame, it is mandatory that the herein-proposed RGs, or those identified in future studies, will not be the pathology-related or disease-influenced target miRNAs to be studied or loaded and eventually verified in their amount with potency assays. At present, for tendon-related diseases, both miR-101-3p and miR-22-5p have not been reported as involved in tissue homeostasis, pathology or healing. If future studies will describe these two molecules involved in tendinopathy and proposed as therapy targets, an update for alternative and effective hAMSC-EVs RGs will be needed, starting from the better positioned ones in the ranking of this manuscript. Regarding their influence on pathological states in general, and vice versa the effect of the pathological states on their expression, both miRNAs have been reported to be involved and differentially expressed in several diseases. For example, miR-101-3p was claimed to be related to kinds of both non-malignant syndromes (multiple system atrophy [75], hepatopulmonary syndrome [76], cardiac fibroblasts [77], HBV-associated chronic hepatitis [78], Alzheimer [79], pulmonary fibrosis [80], acute kidney injury [81], gestational diabetes mellitus [82]) and, especially, malignant neoplasms [83]. Similarly, miR-22-5p is aberrantly expressed in various cancers, such as prostatic cancer [84], esophageal squamous cell carcinoma [85] and breast cancer [86], together with, among others, polyglutamine diseases such as Huntington’s disease [87]. Consistently, searching Vesiclepedia, both miRNAs were reported as embedded in greatly different amounts between EVs from tissue or cancer types. In several reports associated with cancer, both miRNAs were recognized to modulate proliferation, invasion and metastasis by accelerating cell senescence and inhibiting energy metabolism and angiogenesis [83,88]. This means that the environment hAMSCs will be administrated to, and the individuals the amniotic membrane will be collected from, might influence both isolated cells and RG-EV expression. Therefore, to replicate this study and provide a wider picture, further miRNA RG stability experiments will have to be performed with hAMSC-EVs from diseased individuals, or with specific culturing conditions mimicking disease conditions. This will give reliable results to integrate miRNA-EV RG from healthy individuals, as herein reported, and be a future roadmap for basic research—on the cargo of EVs when encountering a pathological environment—and potency assays—release tests for clinical applications—for hAMSCs. Again, if miR-101-3p and miR-22-5p are the studied or loaded molecules in EVs, other RGs will have to be monitored.

## 5. Conclusions

EVs from MSCs have been postulated as cell-free shuttles for therapeutic miRNAs in several clinical settings related to regenerative medicine, such as tendinopathies, where both amniotic allografts, amniotic-derived MSCs and their conditioned medium already showed promising results. Until now, no miRNA therapy has been applied clinically due to the complexity of the target gene networks as well as the efficiency and safety of the delivery system. Overcoming these concerns, biological MSC-EVs might represent a secure and sustainable approach relative to traditional viral systems, although the issue of a deep characterization of their content and their molecular targets remains a challenge. Only the definition of reliable normalizers will allow a correct quantification of embedded molecules, as miRNAs, to develop disease-focused potency assays and compare either donors or cell/tissue sources to make MSC-derived EVs, and hAMSC-EVs in particular, safe and efficacious cell-based drug (miRNA)-delivery platforms for regenerative medicine based approaches. As a milestone, in hAMSC-EVs, miR-101-3p and miR-22-5p resulted as the most stable RGs, with the last being an intriguing candidate to be validated in other MSC types to become a possible miRNA reference gene in therapeutic EVs from mesenchymal stem cells, although, at present, it is not possible to predict its redundancy due to influence on miRNA levels by tissues, individuals and diseases. Therefore, future EV-RG stability analyses will be needed also in other conditions, or with diseased donors for hAMSCs, or with other MSC types.

## 6. Patents

O.P. is author of 2 issued patents (US8524283B2 and EP2171042B1).

## Figures and Tables

**Table 1:** Candidate RGs and tendon-related miRNAs and target sequences.

Accession Number	Gene Name	Target Sequence (5’–3’)	Reference
MIMAT0000062	let-7a-5p	UGAGGUAGUAGGUUGUAUAGUU	[48,49,50]
MIMAT0000069	miR-16-5p	UAGCAGCACGUAAAUAUUGGCG	[51,52]
MIMAT0004495	miR-22-5p	AGUUCUUCAGUGGCAAGCUUUA	[8]
MIMAT0000078	miR-23a-3p	AUCACAUUGCCAGGGAUUUCC	[53]
MIMAT0000082	miR-26a-5p	UUCAAGUAAUCCAGGAUAGGCU	[49,53]
MIMAT0004503	miR-29a-5p	ACUGAUUUCUUUUGGUGUUCAG	[8]
MIMAT0000099	miR-101-3p	UACAGUACUGUGAUAACUGAA	[53]
MIMAT0000101	miR-103a-3p	AGCAGCAUUGUACAGGGCUAUGA	[48]
MIMAT0000278	miR-221-3p	AGCUACAUUGUCUGCUGGGUUUC	[48,49]
MIMAT0004748	miR-423-5p	UGAGGGGCAGAGAGCGAGACUUU	[54]
MIMAT0003393	miR-425-5p	AAUGACACGAUCACUCCCGUUGA	[54]
MIMAT0003338	miR-660-5p	UACCCAUUGCAUAUCGGAGUUG	[8]
NR_004394.1	U6 snRNA	GUGCUCGCUUCGGCAGCACAUAUACUAAAAU UGGAACGATACAGAGAAGAUUAGCAUGGCCC CUGCGCAAGGAUGACACGCAAAUUCGUGAAG CGUUCCAUAUUUU	[55]
*miRNA targets*			
MIMAT0000086	miR-29a-3p	UAGCACCAUCUGAAAUCGGUUA	[56,57]
MIMAT0000428	miR-135a-5p	UAUGGCUUUUUAUUCCUAUGUGA	[58]
MIMAT0000449	miR-146a-5p	UGAGAACUGAAUUCCAUGGGUU	[59]
MIMAT0000754	miR-337-3p	CUCCUAUAUGAUGCCUUUCUUC	[60]
MIMAT0000065	let-7d-5p	AGAGGUAGUAGGUUGCAUAGUU	[59]

**Table 2:** Expression levels of candidate RGs

Gene Name	Genorm M-Value	Normfinder SV	Bestkeeper SD	Delta C_t_ SD	Geomean	Ranking Order
miR-101-3p	0.19 (1)	0.09 (1)	0.18 (2)	0.68 (1)	1.32	1
miR-22-5p	0.19 (1)	0.10 (2)	0.17 (1)	0.69 (2)	1.41	2
miR-221-3p	0.28 (3)	0.24 (4)	0.18 (3)	0.74 (3)	2.91	3
miR-660-5p	0.37 (4)	0.16 (3)	0.26 (4)	0.76 (4)	3.72	4
miR-29a-5p	0.46 (5)	0.66 (5)	0.40 (5)	0.93 (5)	5	5
miR-16-5p	0.59 (6)	0.84 (6)	0.65 (6)	1.03 (6)	6	6
miR-26a-5p	0.66 (7)	1.02 (7)	0.71 (7)	1.13 (7)	7	7
miR-423-5p	0.85 (8)	1.17 (8)	0.81 (8)	1.28 (8)	8	8
U6 snRNA	0.95 (9)	1.18 (9)	0.82 (9)	1.29 (9)	9	9

miRNAs are ranked according to geomean. The numbers in brackets represent the ranking values.
